# Unrelated cord blood transplantation for pediatric patients with inborn error of immunity and inborn error of metabolism in Vietnam: early single-center experience

**DOI:** 10.3389/fimmu.2026.1871545

**Published:** 2026-07-10

**Authors:** Tung Cao-Viet, Le Nguyen-Ngoc-Quynh, Anh Ha-Phuong, Ha Dang-Thi, Binh Nguyen-Thanh, Ngoc Can-Thi-Bich, Dung Vu-Chi, Pamela P. Lee, Chi Le-Quynh, Quynh Bui-Thi-Thuy, Dien Tran-Minh

**Affiliations:** 1 Children Heart Center, National Children’s Hospital, Hanoi, Vietnam; 2 Stem Cells Center, Vietnam National Children’s Hospital, Hanoi, Vietnam; 3 Pathophysiology and Immunology Department, Hanoi Medical University, Hanoi, Vietnam; 4 The Center of Endocrinology, Metabolism, Genetics and Molecular Therapy, Vietnam National Children’s Hospital, Hanoi, Vietnam; 5 Vietnam University of Traditional Medicine, Hanoi, Vietnam; 6 Department of Paediatrics and Adolescent Medicine, School of Clinical Medicine, Li Ka Shing Faculty of Medicine, The University of Hong Kong, Hong Kong, Hong Kong SAR, China; 7 Department of Rheumatology, Allergy, and Immunology, Vietnam National Children’s Hospital, Hanoi, Vietnam; 8 Surgical Intensive Care Unit, Vietnam National Children’s Hospital, Hanoi, Vietnam

**Keywords:** hematopoietic stem cell transplantation, immune reconstitution, inborn errors of immunity, umbilical cord blood, unrelated cord blood transplantation

## Abstract

**Introduction:**

Umbilical cord blood (CB) contains hematopoietic stem and progenitor cells and is an established alternative donor source for hematopoietic stem cell transplantation (HSCT) in both malignant and non-malignant disorders, particularly in children.

**Patients and methods:**

We retrospectively studied 12 consecutive pediatric patients with genetically distinct inborn error disorders who underwent single-unit unrelated cord blood transplantation (UCBT) at Vietnam National Children’s hospital between October 2020 and February 2026. Outcomes were analyzed descriptively, with emphasis on engraftment, graft failure or rejection, graft-versus-host disease (GVHD), infectious complications, donor chimerism, and immune reconstitution.

**Result:**

The median age at transplantation was 19 months (range, 7–42 months), and the median interval from diagnosis to transplantation was 10 months (range, 3–39 months). The study cohort included patients with Wiskott–Aldrich syndrome, very early-onset inflammatory bowel disease caused by an IL10R mutation, severe combined immunodeficiency, major histocompatibility complex class II deficiency, and mucopolysaccharidosis type II. With a median follow-up of 26 months, neutrophil engraftment occurred in nine patients (75%). The cumulative incidence of grade II-IV acute graft-versus-host disease (GVHD) was 41.6%, and no patient developed chronic GVHD. Five patients discontinued immunosuppressive therapy. One patient died due to multiorgan failure within the first month after UCBT. Two patients underwent successful haploidentical transplantation because of graft failure, and one patient developed graft rejection after initial engraftment. No additional deaths occurred during follow-up, resulting in an overall survival rate of 91.1% (11/12), whereas the UCBT success rate was 66.7%. The small sample size and relatively short follow-up for some patients restrict the strength of the study.

**Conclusion:**

UCBT was feasible for children who required stem cell transplantation but lacked a suitable donor, providing sustained donor engraftment and immune recovery in most long-term survivors.

## Introduction

Hematopoietic stem cell transplantation (HSCT) from a healthy donor is an established curative or disease-modifying therapy for a broad range of non-malignant disorders, including inborn errors of immunity (IEIs), hemoglobinopathies, bone marrow failure syndromes, and inborn errors of metabolism (IEMs) (1). In these conditions, transplantation may correct defective hematopoiesis, restore immune function, or provide sustained enzyme replacement through donor-derived hematopoietic cells (1, 2).

Among available donor sources, unrelated umbilical cord blood (UCB) is considered a highly promising donor source for children (2–4). Cryopreserved UCB units are rapidly available, do not require donor mobilization, and may be used despite a greater degree of HLA mismatch than is typically acceptable with grafts from adult donors (2, 4–6). In the past, UCBT was associated with delayed hematopoietic recovery, slower immune reconstitution, and a higher risk of early infectious complications and transplant-related mortality (TRM), especially when the cell dose was low (2, 4, 7).

However, outcomes after UCBT have improved substantially over time. Better public cord blood banking, higher-quality unit selection, increasing attention to total nucleated cell (TNC) and CD34+ cell dose, and advances in supportive care have contributed to faster engraftment, improved immune recovery, and lower TRM (2, 3, 5, 6). In experienced centers, outcomes of UCBT are now comparable with those of other alternative donor sources for selected pediatric indications (1, 2, 7).

Despite these advantages, the use of UCBT has declined over the world, because of the expanding use of haploidentical related donors and modern GVHD prophylaxis strategies (1, 7–9). Nevertheless, UCBT remains an important and sometimes optimal option for pediatric patients who lack a matched related donor and timely access to unrelated adult donors is limited (1–3, 9).

Evidence on UCBT outcomes from low- and middle-income countries remain underreported. We therefore describe the early single-center experience with unrelated single-unit UCBT in children diagnosed with IEIs and IEMs at the National Children’s Hospital over a 5-year period, focusing on feasibility, engraftment, complications, immune reconstitution, and practical lessons learned during implementation.

## Patients and methods

A total of 12 consecutive pediatric patients who met the diagnostic criteria for IEIs or IEMs underwent unrelated single-unit UCBT at the Vietnam National Children’s Hospital between October 2020 and February 2026. Clinical data on patient characteristics, graft characteristics, conditioning, transplantation outcomes, complications, and immune reconstitution were retrospectively collected and analyzed.

Cord blood unit selection was based on a single unrelated unit with high-resolution HLA typing at HLA-A, HLA-B, and HLA-DRB1 (10, 11). A 5/6 or 6/6 matched unit was preferred, with selection also guided by pre-cryopreservation TNC dose and CD34+ cell dose. The target minimum doses were ≥4 × 10^7^ TNC/kg and ≥3 × 10^5^ CD34+ cells/kg recipient body weight, respectively (2, 7, 11).

Conditioning regimens were categorized as myeloablative conditioning (MAC) or reduced-intensity conditioning (RIC). Regimens containing busulfan at a total dose of ≥12 mg/kg or melphalan at a total dose of ≥150 mg/m^2^ were classified as MAC, consistent with Center for International Blood and Marrow Transplant Research criteria and prior studies (12). RIC regimens are defined as conditioning approaches that use less than 8 Gy of total body irradiation, less than 8 mg/kg oral busulfan or an equivalent intravenous dose, or alternative agents with potent immunosuppressive activity but lower tissue toxicity than conventional myeloablative regimens (12). This approach may reduce acute graft-versus-host disease and other early transplant-related complications, particularly in patients with significant pretransplant comorbidities or infection burden. Conditioning and supportive care were protocol based rather than disease specific, with individualized adjustment according to infection status, organ function, and clinical conditions.

GVHD prophylaxis consisted primarily of intravenous cyclosporin (target trough level, 150–200 ng/mL) and mycophenolate mofetil (MMF) 15 mg/kg every 8 h starting on day −2 (10). Cyclosporin was switched to twice-daily oral administration once the patient was able to tolerate oral intake. In patients without active GVHD, MMF was discontinued after day 28, and cyclosporin tapering was initiated from day 100 onward. Patients with active viral infection were allowed a more rapid MMF taper. Acute GVHD was graded according to standard consensus criteria (13).

Before conditioning, patients were admitted to single rooms with high-efficiency particulate air filtration, with one guardian allowed to stay with each patient. Hickman central venous catheters were inserted to provide therapeutic access. Healthcare personnel were required to follow strict hand hygiene practices and use personal protective equipment, including gowns, gloves, and masks. Antifungal prophylaxis consisted of intravenous micafungin followed by oral voriconazole near discharge. Antibacterial prophylaxis was initiated on day −3. Patients at risk for *Herpes simplex virus* or *Varicella-zoster virus* received acyclovir from day 0. *Cytomegalovirus* (CMV) and *Epstein–Barr virus* (EBV) monitoring was performed weekly. Patients who were CMV-seropositive and/or CMV polymerase chain reaction (PCR) positive received ganciclovir prophylaxis from day −12 to day −1, followed by acyclovir or foscarnet. Patients with a history of EBV viremia before transplantation received a single dose of rituximab on day −1 to reduce the risk of post-transplant EBV reactivation. *Pneumocystis jirovecii* prophylaxis with trimethoprim-sulfamethoxazole was given before transplantation, discontinued on day 0, and restarted after neutrophil engraftment. Intravenous immunoglobulin (500 mg/kg) was administered when the serum IgG level was <500 mg/dL. Ursodiol was used for sinusoidal obstruction syndrome prophylaxis.

Cord blood grafts were infused over 15 to 30 min without thawing and dilution. Neutrophil recovery was defined as achievement of an absolute neutrophil count ≥0.5 × 10^9^/L for 3 consecutive days. Platelet recovery was defined as the first day of a platelet count >20 × 10^9^/L without platelet transfusion in the preceding 7 days. Graft failure was defined as the lack of hematopoietic cell engraftment following allogeneic stem cell transplantation. Graft rejection refers to the immunologic rejection of donor hematopoietic elements by the residual host immune system.

Chimerism was assessed in whole blood using short tandem repeat analysis, and peripheral blood lymphocyte reconstitution was monitored by flow cytometry. Immune reconstitution was evaluated before conditioning and serially after transplantation and was compared with the lower limit of the age-adjusted normal range for CD3, CD4, CD8, CD19, and natural killer (NK) cell counts.

This is a descriptive study carried out in HSCT center at the Vietnam National Children’s Hospital. Data were summarized using medians and ranges for continuous variables and counts and percentages for categorical variables. Because of the small cohort size and the inclusion of several different diagnoses, descriptive comparison according to HLA match, CMV status, or CD34+ cell dose are used. The primary endpoint was overall survival (OS), defined as survival regardless of disease status. Secondary endpoints included cumulative incidence of neutrophil recovery, acute and chronic GVHD, and TRM. All analyses were performed using IBM SPSS Statistics version 26.0.

### Ethical approval and informed consent

All procedures involving human participants were performed in accordance with institutional and national ethical standards. All patients or their parents/legal guardians provided informed consent for treatment, data collection, analysis, and publication. As a retrospective descriptive study, this study adhered to the ethical principles of biomedical research.

## Results

### Patients and cord blood unit characteristics

Among the 12 patients who underwent transplantation, seven (58.4%) were diagnosed with Wiskott–Aldrich syndrome, one (8.3%) with severe combined immunodeficiency, one (8.3%) with major histocompatibility complex (MHC) class II deficiency, and one (8.3%) with mucopolysaccharidosis type II. The remaining two patients (16.7%) had very early-onset inflammatory bowel disease associated with IL10R mutations, including one case with an IL10RA mutation and one with an IL10RB mutation (**Tables 1**, **2**). Several individual cases from this cohort have been reported previously (14–17). The median age at transplantation was 19 months, 11 patients (91.7%) were men, and 7 patients (58.4%) were CMV PCR-positive before transplantation. The median interval from diagnosis to UCBT was 10 months. Notably, waiting time from diagnosis to UCBT decreased from 18–39 months before 2024 to 3–6 months after 2024 (data not shown).

**Table 1:** Patients and graft characteristics (n=12).

Variables	Value
Patient characteristics	
Age at transplant, median (range), months	19 (7- 42)
Time from diagnosis to transplantation, median (range), months	10 (3-39)
Male sex, n (%)	11 (91.7)
Indication for transplant, n (%)	
Wiskott–Aldrich syndrome	7 (58.4)
Severe combined immunodeficiency disease	1 (8.3)
Major histocompatibility complex class II deficiency	1 (8.3)
Mucopolysaccharidosis type 2	1 (8.3)
Very early-onset inflammatory bowel disease	2 (16.7)
Recipient CMV status at transplant	7 (58.4)
Graft characteristics	
High resolution HLA match (of 6), n (%)	
5/6	7 (58.3)
6/6	5 (41.7)
Donor-specific anti HLA antibodies, n (%)	1 (8.3)
Infused TNC/kg, median (range), x 10^7^	8.07 (6.6-13.57)
Infused CD34/kg, median (range) x 10^5^	6.92 (3.27-12.23)
Blood group ABO mismatch, n (%)	3 (25)
Conditioning regimen	
Busulfan-containing regimen	12 (100)
Cyclophosphamide	3 (25)
Fludarabin	11 (91.7)
Anti-thymocyte globulin	11 (91.7)
GVHD prophylaxis	
Cyclosporin + mycophenolate mofetil	10 (83.4)
Cyclosporin	1 (8.3)
Cyclosporin + corticosteroid	1 (8.3)

CMV, cytomegalovirus; HLA, human leukocyte antigen; TNC, total nucleated cell; CD34+, stem cell; GVHD, graft-versus-host disease.

**Table 2:** Clinical manifestations and complications of patients with inborn error of immunity before transplant (n=11).

Category	Total cases	Male gender	Age at onset (mo)	Age at diagnosis (mo)	Family history (no. of cases)	Recurrent respiratory infection (no. of cases)	Recurrent diarrhea (no. of cases)	Skin infection (no. of cases)	Fungal infection (no. of cases)	Meningitis (no. of cases)	BCG osis (no. of cases)
SCID	1	0	2	5.5	0	1	1	0	1	0	1
WAS	7	7	1.00 ± 0.14 (0.5-2)	3.5 ± 1.98 (2-6)	0	7	5		2	0	0
VEO-IBD	2	2	1	6	0	1	2	1	1	0	0
MHC II	1	1	4	5	0	1	1	0	0	0	1
Total	11	10			0	10	9	1	4	0	2

BCG, Bacillus Calmette–Guérin; SCID, severe combined immunodeficiency; WAS, Wiskott–Aldrich syndrome; VEO-IBD, very early onset inflammatory bowel disease; MHC II, major histocompatibility complex class II deficiency.

All patients received MAC conditioning regimen. Donor-specific anti-HLA antibody test results were negative in 11 patients (91.7%).

Seven patients (58.3%) received a high-resolution 5/6 HLA-matched cord blood unit, and five (41.7%) received a 6/6 matched unit. In this cohort, both the infused TNC dose and CD34+ cell dose were relatively high, with medians of 8.07 × 10^7^ and 6.92 × 10^5^/kg, respectively (**Table 1**). Three grafts were major ABO-mismatched.

### Engraftment and overall survival

Neutrophil engraftment occurred in 9 of 12 patients. The median time to neutrophil and platelet engraftment was 15 days (range, 11–25 days) and 35 days (range, 25–57 days), respectively. In this small cohort, descriptive comparisons did not show a clear pattern between the degree of HLA mismatch and time to neutrophil engraftment (**Table 3**). We observed a faster neutrophil recovery with higher CD34+ cell dose; however, this difference is not statistically significant, likely because of the limited sample size. No significant association was observed between cell dose and platelet engraftment.

**Table 3:** Neutrophil and platelet engraftment post-transplant (n=9).

Characteristics	Neutrophil engraftment (day)	Platelet engraftment (day)
	X ± SD	X ± SD
HLA mismatch:		
5/6	16.3 ± 6.1	37.5 ± 15.2
6/6	16.3 ± 3.1	34.7 ± 3.5
CMV:		
Negative	12.0 ± 1.4	32 ± 8.5
Positive	18 ± 4.3	38 ± 12.3
CD34+ × 10^5^/kg:		
3-5	25	35
>5	14.8	42

CMV, cytomegalovirus; HLA, human leukocyte antigen; CD34+, stem cell.

The median follow-up among survivors was 26 months (range, 2-62 months). One patient died of organ failure within the first month after UCBT, resulting in an overall survival of 91.7% (11/12 patient). Two patients (a Wiskott–Aldrich patient and a mucopolysaccharidosis type II) experienced primary graft failure after UCBT and subsequently underwent successful rescue haploidentical transplantation within 2 months using reduced-toxicity conditioning (14, 17). An IL10RB patient developed graft rejection 40 days post-transplant, and he is scheduled for a second transplant. When death, primary graft failure, and graft rejection were counted as events, UCBT event-free survival was 66.7% (8/12 patient).

**Figure 1:**
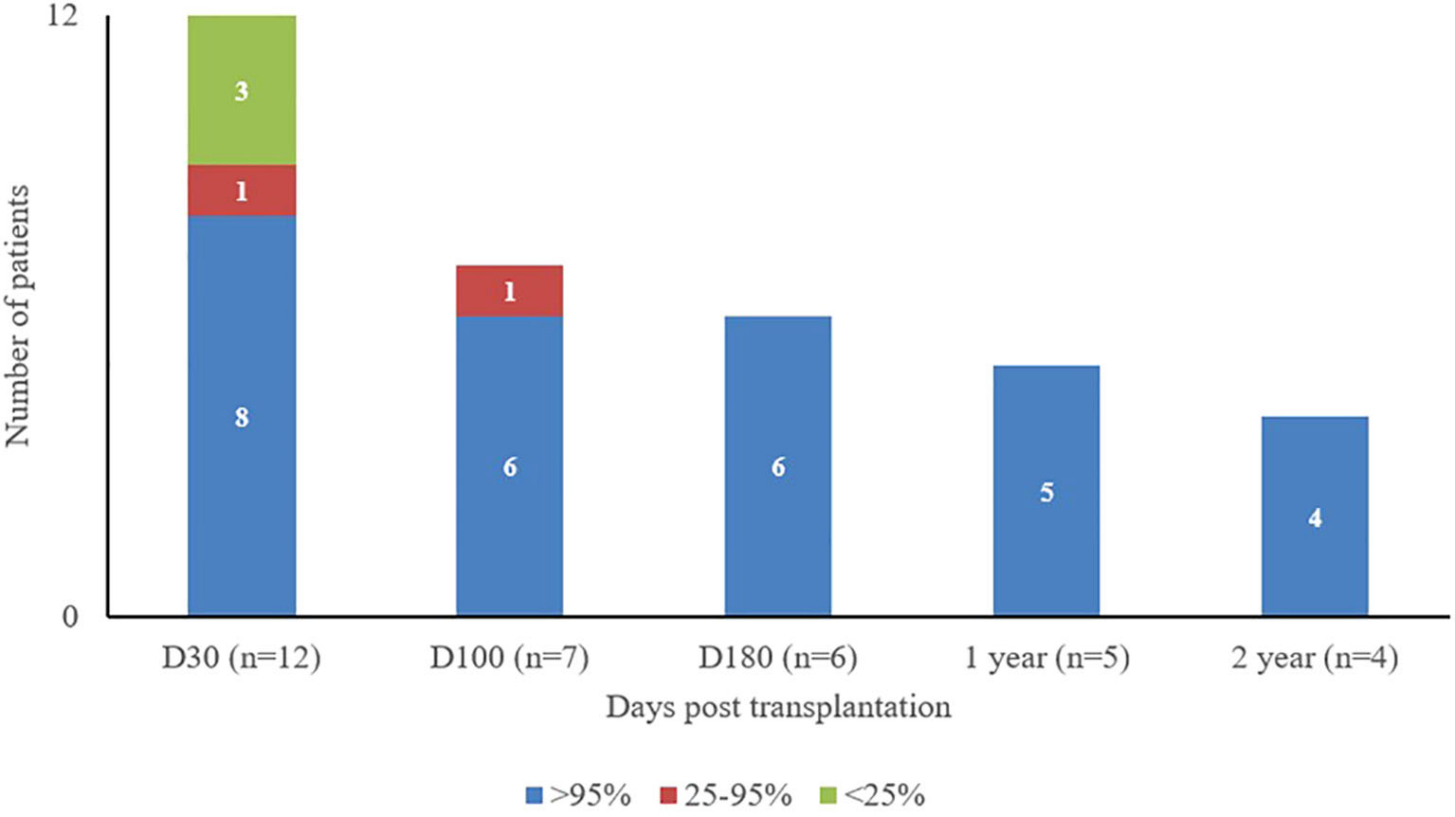
Donor chimerism after unrelated cord blood transplantation. Bar chart showing the distribution of whole blood chimerism at day 30, day 100, day 180, 1 year and 2 years post transplantation. Chimerism was categorized as >95%, 25-95% and <25%.

Whole-blood donor chimerism was ≥95% in 8 of 12 patients on day 30, 6 of 6 patients on day 180, 5 of 5 patients on 1 year, and 4 of 4 patients at 2 years after transplantation (**Table 4** and **Figure 1**). These later time-point estimates should be interpreted cautiously because they were based on progressively small numbers of patients.

**Table 4:** Post-transplant complications and chimerism results (n=12).

Complications	Value
Engraftment syndrome, n (%)	7 (58.3)
Acute GVHD, n (%)	5 (41.6)
Grade 1	3 (25)
Grade 2	0 (0)
Grade 3	1 (8.3)
Grade 4	1 (8.3)
Whole blood chimerism at 1 month (n=12), n(%)		
>95%	8 (66.7)
25-95%	1 (8.3)
<25%	3 (25)
Opportunistic infections at 1 year	
CMV	6 (50)
EBV	2 (16.7)
HHV6	1 (8.3)
Norovirus Rotavirus	1 (8.3) 1 (8.3)
Bacteria	1 (8.3)
Fungal	1 (8.3)

CMV, cytomegalovirus; EBV, Epstein–Barr virus; HHV6, human herpex virus; GVHD, graft-versus-host disease.

### Acute and chronic GVHD

The cumulative incidence of acute GVHD by day 100 was 41.6%. Three patients had grade I acute GVHD, and two patients had grade III-IV acute GVHD. No patient developed chronic GVHD during follow-up. Because of small cohort, no definitive association between acute GVHD and HLA match, ABO compatibility, or post-transplant CMV reactivation could be inferred (**Table 4**).

### Transplant-related complications

The cumulative incidence of first opportunistic infection within 1 year after transplantation was 50%, with a median onset of 8 days after transplantation (range, day −2 to day 36). Eight patients developed infections caused by more than one pathogen (**Table 4**). The most common event was asymptomatic CMV viremia, which was managed with appropriate antiviral therapy. Within 100 days after transplantation, viral gastroenteritis was observed in two patients (16.7%), caused by rotavirus and norovirus. One patient developed invasive *Trichosporon asahii* infection on day 24 (17), and one patient developed *Klebsiella aerogenes* septicemia and died of multiorgan failure (15).

No patient developed transplant-associated thrombotic microangiopathy, interstitial pneumonitis, autoimmune hemolytic anemia, pericardial effusion requiring intervention, sinusoidal obstruction syndrome, macroscopic hematuria, or hemorrhagic cystitis. Overall, regimen-related toxicity was limited.

### Immune reconstruction

**Figure 2:**
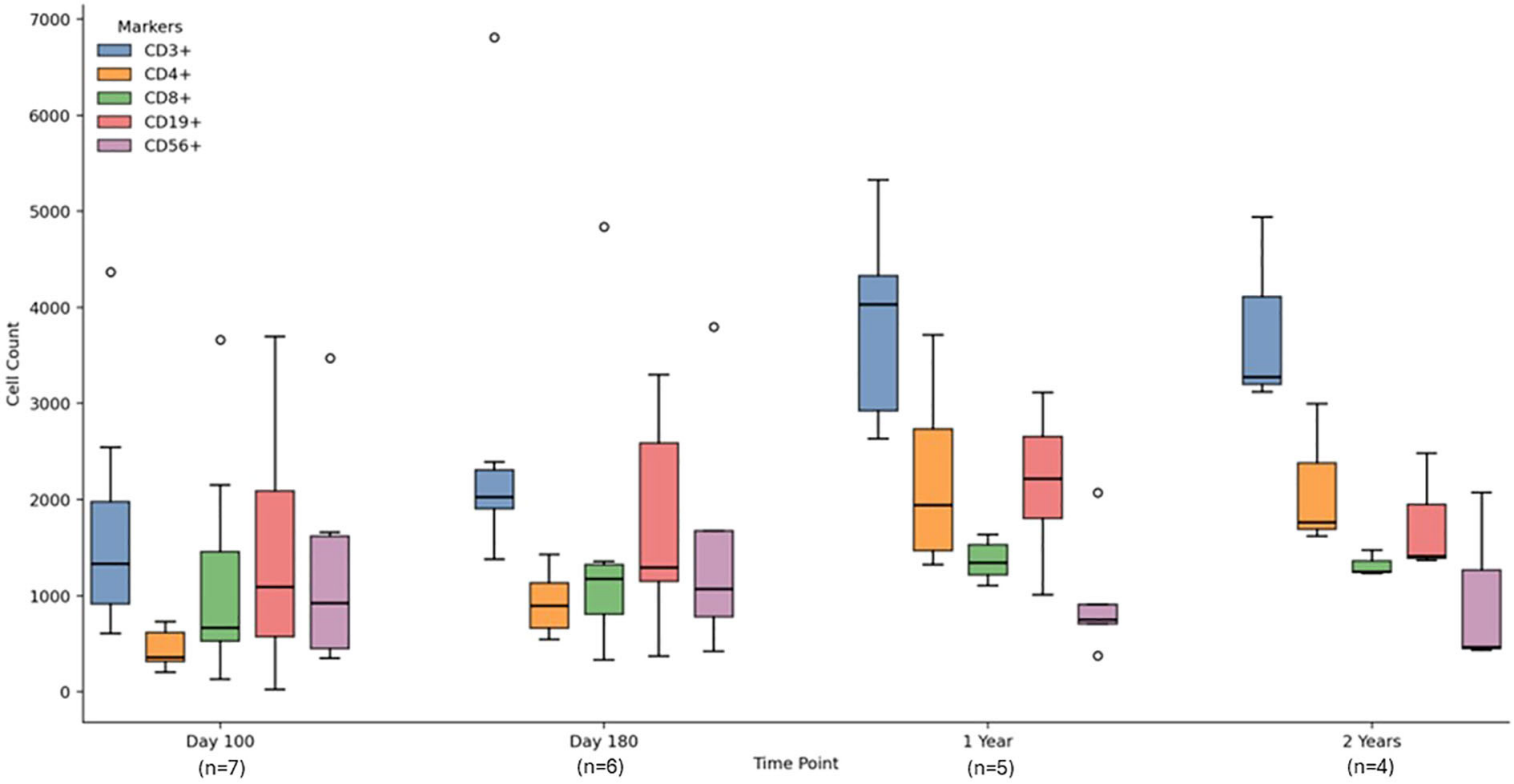
Immune reconstitution after unrelated cord blood transplantation Box plots showing peripheral blood lymphocyte subset counts, including CD3+, CD4+, CD8+, CD19+, CD56+ cells at day 100, day 180, 1 year and 2 years after transplantation.

Immune reconstitution followed the expected post-UCBT pattern. NK-cell and T-cell counts recovered to the age-adjusted normal range by day 100 after transplantation, whereas B-cell reconstitution was slower and accelerated between day 100 and day 180 (**Figure 2**). This immune recovery pattern is clinically relevant in children with IEIs because delayed adaptive immune reconstitution contributes to vulnerability to opportunistic infections in the early post-transplant period. In children with inborn errors of immunity, this information is clinically important because immune recovery is directly linked to infection risk, discontinuation of immunosuppression, functional recovery, and long-term transplant success.

## Discussion

The prevalence of IEIs and IEMs has increased in recent years, due to advances in newborn screening and improved clinician awareness of these disorders. HSCT remains the main curative treatment for most IEIs; however, for IEMs, the roles of HSCT and gene therapy remain controversial and are still evolving. This single-center study described the early single-center experience with unrelated single-unit CBT in children with IEIs and IEMs over a 5-year period. The overall survival rate of 91.7% in our cohort indicates that UCBT is a feasible alternative donor strategy in case-matched related donors which are not often available. The most important contribution of this study is the documentation of feasibility, early outcomes, immune reconstitution, and implementation lessons from a Vietnamese pediatric transplant center. To the best of our knowledge, this is the first report on this topic from Vietnam. Our results should be interpreted in the context of a heterogeneous case report and a small sample size, but they supported the continued role of UCBT in carefully selected pediatric patients (1–4). The study included children with different underlying disorders, including WAS, SCID, MHC class II deficiency, VEO-IBD caused by an IL10R mutation, and MPS II. This diversity reflects real-world referral patterns, but it prevents meaningful disease-specific outcome comparisons. Accordingly, the present analysis was framed as a descriptive case series rather than a statistically powered efficacy study.

In children, identifying a UCB unit with an adequate cell dose is often feasible because of lower body weight (18). A suitable single-unit cord blood graft was identified for all 12 eligible children, highlighting the broad utility of cord blood even across HLA allele mismatch. The median infused TNC and CD34+ cell doses were above commonly recommended thresholds (2, 7, 11). This also suggested that unmanipulated single-unit UCB may be a practical and cost-effective option for children with IEI and IEM disorders, instead of other additional supportive graft strategies such as double-cord or haplo-cord transplantation. Cell dose remains a critical determinant of engraftment after UCBT, and current selection strategies emphasize the combined importance of TNC/CD34+ dose and HLA matching (2, 10, 14, 19). In our cohort, although descriptive data suggested earlier neutrophil recovery among patients who received higher CD34+ cell doses, this observation should be interpreted cautiously given the small sample size. Larger studies have demonstrated the combined importance of cell doses and HLA matching for UCBT outcomes (7, 11, 18, 19). The interaction between cell doses and HLA matching has been extensively studied. Barker et al. (7) demonstrated a combined effect of TNC dose and HLA match on transplantation outcome, whereas Eapen et al. (20) and Kurtzberg et al. (18) showed that UCBT can achieve favorable long-term outcome in pediatric transplantation. More recently, Eapen et al. (20) reported that allele-level HLA matching is associated with improved survival and lower graft failure after single-unit UCBT, particularly in non-malignant disorders. Our findings were directionally consistent with these data, although our cohort was too small to detect statistically significant differences across matching strata.

Among patients who achieved engraftment in this study, the median time to neutrophil recovery was 15 days, which compares favorably with previously reported pediatric UCBT series (2–4). The primary graft failure rate was 16.7%, slightly higher than that reported in some larger series (18), but these two patients were successfully salvaged with haploidentical transplantation. One patient (8.3%) with IL10R deficiency experienced graft rejection 2 months after transplantation and is being considered for a second transplant. These findings highlight the importance of close early chimerism monitoring and advance planning for rescue donor options, especially in children with IEIs, for whom stable donor engraftment and immune recovery are central therapeutic goals. Importantly, regimen-related toxicities were limited, and no patient developed sinusoidal obstruction syndrome, hemorrhagic cystitis, transplant-associated thrombotic microangiopathy, or severe cardiopulmonary toxicity.

The OS of 91.7% should be interpreted together with UCBT event-free survival of 66.7%. The distinction is important: most patients survived, including those who had been rescued with subsequent haploidentical transplantation, but one-third experienced death, primary graft failure, or graft rejection as UCBT-related events. Reporting both outcomes provides a more balanced interpretation than OS alone and is particularly relevant for counseling families and planning transplant programs.

Acute GVHD occurred in 41.7% of patients, which is within the range reported in previous cord blood studies (8, 18, 19). Consistent with prior literature, chronic GVHD was uncommon and was not observed in our cohort (8, 9, 18, 19). Because of the limited sample size, we could not determine whether HLA disparity, ABO mismatch, or CMV reactivation influenced GVHD risk.

Infectious complications remain a major challenge after UCBT particularly in the early posttransplant period because of delayed engraftment (20, 21). In our cohort, viral reactivation particularly CMV viremia was common, but most episodes were manageable with surveillance and preemptive therapy. This pattern is consistent with previous reports showing that UCBT recipients are vulnerable to early opportunistic infections whereas adaptive immunity gradually recovers over the first several months after transplantation (1, 2, 8).

Immune reconstitution in our cohort followed the expected pattern, with earlier recovery of NK cells and T cells and later recovery of B cells (22, 23). This finding is clinically relevant because timely immune recovery is closely linked to infection risk, survival, and functional recovery after transplantation in children (3, 18). In children with IEIs and other non-malignant disorders, immune recovery is not only a laboratory outcome but also a clinically meaningful marker linked to infection risk, discontinuation of immunosuppression, functional recovery, and long-term transplant success.

Several practical lessons emerged from this early experience. First, careful cord blood unit selection remains essential, with priority given to adequate TNC and CD34+ cell doses, high-resolution HLA matching, and absence of clinically relevant donor-specific anti-HLA antibodies. Second, early and serial chimerism monitoring is critical to identifying impending graft failure or rejection and to allow timely planning for rescue transplantation. Third, because infectious complications are frequent after UCBT, transplant programs should strengthen viral surveillance, preemptive treatment protocols, antifungal strategies, and multidisciplinary infectious-disease support. Fourth, in less-resourced settings, UCBT may remain a practical option when unrelated adult donors are difficult to access, but a backup donor strategy, often haploidentical, should be discussed before conditioning. Finally, reducing the interval from diagnosis to transplantation may improve patient condition at transplant and reduce pretransplant infectious burden.

This study has several limitations, including its retrospective design, single-center setting, small sample size, and relatively short follow-up for some patients. In addition, we did not classify different subtypes of IEIs due to small sample size for each subtype, which limits disease-specific inference. Larger multicenter studies are needed to define the optimal role of UCBT compared with other alternative donor approaches in children.

## Conclusion

In conclusion, our experience suggests that single-unit UCBT is a feasible option for children with non-malignant disorders who lack a matched related donor. In this cohort, UCBT provided acceptable engraftment, limited long-term toxicity, and encouraging survival. Once an adequate cell dose is secured, careful attention to HLA matching, supportive care, infection monitoring, and post-transplant follow-up remains essential to optimize outcomes.

## Data Availability

The raw data supporting the conclusions of this article will be made available by the authors, without undue reservation.
